# On the Mastery of Elementary Geometric Concepts^¤^

**DOI:** 10.11621/pir.2023.0301

**Published:** 2023-09-15

**Authors:** Nina F. Talyzina

**Affiliations:** Lomonosov Moscow State University, Moscow, Russia

Our research is part of a cycle of works on the study of mental actions undertaken by P.Ya. Gal’perin. All the studies of this cycle were performed with a variety of specific materials: arithmetic, algebraic, grammatical, etc. The differences in the material allow us to discover different aspects of the general process of mastering knowledge. Geometry, combining a high degree of abstraction with the visual and concrete, is of particular interest from this standpoint.

We took elementary geometric concepts as the subject of our research: “line,” “angle,” “angle bisector,” “perpendicular,” “adjacent angles,” and “supplementary angles.”

To identify how these concepts are mastered in the process of academic instruction, we first performed an *observational experiment.*

The mastery of concepts was studied as they were applied to solving various geometric problems. The problems varied in several of their attributes:

1. The degree of complexity: in some it was required to use several of the concepts of interest to us, and in others only one; in some, a geometric phenomenon was presented in isolation, and in others it was included in one system of other geometric phenomena or another, some known and some unknown to the subjects;
2. The completeness of the problem conditions: some problems were presented with sufficient conditions, others had missing ones;
3. The conditions specified in the problem conditions and the illustration: in some, the illustration corresponded to the conditions, and in others it contradicted them.

The problems in which the illustration corresponded to the problem conditions were presented first without an illustration, and then with one. Finally, the subjects had to solve the problems silently, and only if they encountered a difficulty were they allowed to speak aloud or in a whisper.

The experiments were performed with 22 students of grades 6–9 who had different degrees of proficiency in geometry.

In the observational experiment, first of all, *difierent levels of mastery of the geometric concepts were established.* The results generally confirm the process of mastering knowledge that was found in earlier studies of this research cycle, but they can serve to characterize in greater detail the levels established in those studies.

Second, a number of defects in the mastery of knowledge were found that had been repeatedly noted in the works of N.A. Menchinskaia, E.N. Kabanova-Meller, V.I. Zykova, and others. The main ones are these:

1. Great unevenness in the mastery of concepts, both within one grade and across different grades.
2. The majority of subjects in grades 6–7 do not know how to use necessary and sufficient attributes to establish whether a particular geometric phenomenon is present under the conditions specified in a given problem. As a rule, these subjects give correct definitions; that is, they know the necessary and sufficient attributes, but when solving problems they do not rely on them, but rather on a visual image of the relevant geometric phenomenon.
3. A significant proportion of sixth graders do not know how to construct an illustration that accords with both the definition given to them and with the problem conditions; they also cannot pick out the elements specified in the problem conditions on the illustration.

All these facts were of auxiliary importance for us: they allowed us to establish the directions along which various properties of the concept to be mastered should be developed.

*The training experiment* was aimed at forming complete geometric concepts and identifying their influence on the formation of further geometric concepts. Taking into account the data obtained in P.Ya. Gal’perin’s studies on the formation of mental actions and concepts, as well as the results of the observational experiment, we devoted special attention to the organization of action to apply attributes of concepts to the solution of various geometric problems.

This was expressed as follows.

First, from all of the attributes to be mastered, we singled out those that were necessary and sufficient to determine the presence or absence of the relevant phenomenon in the material presented.

Second, these attributes were given initially in a “materialized” visual form (written out on a card), then in a verbal form (spoken aloud by the subject), and only after that did the subject use them silently.

Third, we explained how to apply the attributes of a concept and asked for a consistent and undeviating application of all these attributes to the material presented.

Fourth, the problems were specially selected: not only were problems with sufficient conditions and a suitable illustration presented for the pupils to solve, but also various problems with missing conditions and differences between orally formulated conditions and drawings.

Considerable attention was also paid to the subject’s depiction of various geometric phenomena in accordance with orally presented conditions, and the identification of orally presented phenomena both on the illustration and on models of some of the geometric figures.

Two pupils were chosen as subjects. One of them, a sixth grader who was not doing well, was unable to solve a single problem in the observational experiment. She was described by her mathematics teacher as hopeless. The second subject was a fifth grader (who was not studying geometry), who had a grade of “three” [satisfactory — translator’s note] in arithmetic.

The first concept that we formed in our subjects was that of “straight line.” As a defining attribute, we compared a straight line with a taut thread. Alongside the concept of “straight line,” we introduced the concepts of “curved line” and “broken line.” The subjects were given a thread and a card on which the attributes of these lines were written out and illustrations of them were provided.

Thirty problems were given to them to solve. In the problem conditions, either attributes were given that corresponded to one of the lines, or a task was formulated that the subjects had to perform on the proposed model or illustration (to determine, for example, which lines make up a triangle, the letter *a, u*, etc.

Furthermore, problems were introduced in which the illustration contradicted the problem conditions or contained only one of the types of lines that met the given conditions. Several problems were presented with no illustration.

When solving problems for which the conditions described attributes of a line, we asked the subjects to use cards on which the attributes were written out, but in problems that required determination of the type of line on a model or illustration, the subjects used only a thread. It should be noted that starting already with the second problem, the subjects tried to “eyeball it,” but the experimenter kept forcing them in the first 10 problems to use the card or thread.

After receiving the answer, we asked the subjects to substantiate it (if they had not already done so on their own) and to point out the corresponding geometric shapes in the illustration. In problems where the illustration did not match the problem conditions, we asked for a picture that would match the conditions.

After the first 10 problems were solved, we introduced the subjects to the properties of a straight line: a) only one straight line can be drawn between two points, b) a straight line is the shortest distance between two points. These attributes were used in solving subsequent problems.

When solving all the other problems, the subjects, following our instruction, first spoke these attributes aloud, and then used them silently.

Subject K. (fifth grader) solved 28 out of the 30 problems quite correctly, and each time she defended her solution, referring to the problem conditions. Here are some examples.

Problem No. 7. “The girl drew a line on the paper with blue ink. Then she took a red thread, laid it on the line, and stretched it out. When she asked her brother what he saw, he said he saw a red thread. Why didn’t the brother see the line the girl had drawn in ink?” The illustration showed a straight line.

Subject: It’s a straight line because a straight line aligns with the thread.

Experimenter: And if a curve had been drawn, how would you solve the problem?

Subject: It would be drawn incorrectly. It’s a straight line.

Problem No. 25. “A pedestrian and a cyclist are moving between points A and B. The pedestrian takes the shortest route, but the cyclist takes a detour because the road is bad. Along what lines did the travelers move?”

The subject reads the problem to herself and immediately says: “The pedestrian walked in a straight line, but the cyclist — we don’t know: maybe along a curve, maybe along a broken line.”

Experimenter: Or maybe in a straight line?

Subject: The pedestrian goes in a straight line, since it says that he is taking the shortest route, and there can only be one straight line between two points. That means the other one is not going in a straight line.

Two of the problems gave this subject some trouble. Under the conditions given for these problems, it was possible to establish only the absence of attributes of a straight line. It was still necessary to determine what type of line was shown. The answer should have been: a curve or a broken line. The subject at first gave only one of those as an answer. She added the second possible option only after an additional question from the experimenter.

It should be noted that in one of these problems, the inaccuracy of the answer was apparently because the word “twisted” was used in the problem condition. The subject took this to be an attribute of a curved line. Asked by the experimenter why she believed that the route follows a curved line, the subject answered: “the route *twisted.*”

Subject N. (sixth grader) also solved the absolute majority of the problems correctly right away. In four of the problems, when solving them silently to herself, she first gave incorrect answers. In these cases, the experimenter asked her to speak aloud the attributes of the line and to look again at the problem conditions. After that, the subject gave the correct solution for all of the problems.

The second concept we developed was that of “angle.” Unlike the concept of “straight line,” the angle contains two attributes: a) two rays; b) emanating from one point.

The preliminary dialogue showed that subject K. (fifth grader) was only ab le to draw an angle, whereas subject N. (sixth grader) knew the definition of an angle and drew it correctly. However, she could not identify the attributes necessary to establish the presence of an angle. In our preliminary explanation, we emphasized that an angle can only exist when both attributes are present in the problem conditions. Cases were analyzed when only one of the attributes was present. The way we approached formation of the concept was in general the same as for the concept of “straight line,” but with half as many problems. The attributes were also first written out on a card, then spoken aloud, and finally, silently.

As in the previous series, the subjects immediately solved the vast majority of problems correctly, and they easily substantiated their solutions, stating the missing attributes without difficulty.

An example was the solution of *problem No. 7* by subject N. (sixth grader), in which two arcs intersect at point *M.* The subject is required to figure out what shapes they form. The illustration shows arcs that are close to being straight lines.

Subject: These are not angles: you need to have rays — those are straight lines — and they have to intersect or proceed from one point, but these are arcs — curves — which means there are no angles.

Experimenter: What attribute is there that is for angles?

Subject: A point in common.

Experimenter: What is missing?

Subject: These are curves, but we need straight lines.

A similar thing happened in solving other problems.

The subjects made mistakes in several cases. Subject K. (fifth grader) gave the wrong answer to three problems. In one of them, two rays and a point were presented, but it was not said that the rays emanate from that point. The subject did not notice this and answered that there was an angle. After being prompted to check again, the error was fixed.

In the other two problems, an error was made because the attributes were presented in an indirect form. Thus, in one of the problems, two intersecting lines were shown. It was necessary to establish whether the resulting figures would be angles. Since we had not familiarized the subject with the intersections of lines, she was not able to establish the existence of a common point from the information she was given. “It doesn’t say anything about a point,” says the subject. Characteristically, the subject did not consider the presence of angles in the illustration. She was looking for attributes of an angle in the problem conditions.

Subject N. (sixth grader), as in the previous series, made several mistakes while solving the problems silently. But in all cases, when she went back to the attributes, repeating them aloud, she independently corrected the error. Thus, for example, in one of the problems a point *A* and two rays were shown. The question was what figure was formed. The illustration depicts an angle. The subject says there is an angle. She explained it like this: “Point *A* — this is a vertex, two rays come out of it. And if the rays come from one point, then an angle is formed.” The experimenter suggests naming the attributes of an angle and looking to see if they are all there in the problem conditions. The subject detects an error: “The illustration is wrong; the problem conditions do not say that the rays come *from one* point.” The experimenter asks for another illustration that matches the problem conditions. The subject separately draws point *A* and two rays.

*In this way, by objectifying the attributes of concepts and showing a method of acting with them, the vast majority of problems requiring the use of these attributes were solved correctly by the subjects. If a wrong solution was chosen, the use of attributes in an objectified form allowed the subjects to independently correct the mistake.*

When forming the next concept — “angle bisector” — we decided to see whether the method of action would be transferred from one concept to another. To this end, when forming the concept of “angle bisector,” we did not conduct a stage-by-stage development of attributes. With subject K. (fifth grader), we drew the bisector of an angle and gave a definition. Subject N. (sixth grader) did it on her own. We asked the subjects to identify the attributes by which one can learn whether the problem conditions describe a bisector. Both subjects were able to do it. After that, the subjects were asked to solve the problems silently right away. The problems to be solved were taken from the observational experiment, requiring the use of the concept of “angle bisector.” Subject K. (fifth grader) solved them for the first time. Subject N. (sixth grader) had dealt with them in the observational experiment, but had not solved any of them.

All the problems were solved quickly and correctly by both subjects, and subject N. (sixth grader) solved five out of six silently, and solved only one aloud, in which the attributes of the bisector were included in a system of geometric concepts unfamiliar to her.

Subject K. (fifth grader) solved all the problems silently, and she was given not six, but eight.

As an illustration, we will compare the solution of two problems by subject N. (sixth grader) in both the observational and training experiments.

*Problem No. 2.* “In a triangle, a straight line is drawn from the vertex to the base so that it divides the angle at the vertex into two equal parts. Will this line be the bisector of the angle at the vertex?” An illustration was provided, in which the triangle is indicated by the letters *A, B,*and *C.* The bisector of the angle at the vertex is indicated by the letters *B* and *D.*

*The observational experiment.*

Long pause.

Subject: It will be.

Experimenter: Why?

Subject: Because it divides the triangle into two equal angles.

Experimenter: Name these angles.

Subject. *AB... AD...*No (she can’t name them).

Experimenter: Name the line that bisects the angle.

Subject: Line *B... BD* (names it correctly).

Experimenter: What equal angles did it form?

Subject: *BDC* (wrong).

Experimenter: Show it with a pencil (subject can’t do it). Which angle did it divide into two equal parts?

Subject: circles the letters *A, B,* and *D.*

We see that the subject cannot name and point out the angle in which the bisector is drawn and the angles it forms. Furthermore, she does not connect the angle in which the bisector is drawn with the angles formed after the division: she points out the bisector correctly (in the angle at the vertex), but shows the angles it forms at the point of intersection of the bisector with the base of the triangle. When the experimenter then asked again why she believed that *BD* is a bisector, the subject answered: “It divides the angles, which means it is straight at the vertex, so it is a bisector.”

We see that while at first the subject indicated one attribute of a bisector: “It divides the triangle into two equal angles,” now she indicates a completely different attribute: “It divides the angles, is straight at the top.” She does not identify the necessary and sufficient attributes of a bisector, although she gives the correct definition.

*In the training experiment,* this is how she solved the same problem:

Reads the problem to herself. Traces the line with her finger. Begins to list aloud the attributes of a bisector. The experimenter interrupts: “Have you solved the problem yet?”

Subject: I solved it. It is a bisector.

Experimenter: Why?

The subject correctly names the attributes of a bisector, indicates their presence in the problem conditions, correctly shows the angles that are formed.

Another example. *Problem No. 5.* A bisector is given in the problem conditions, but the illustration shows a straight line, in a position quite different from that of a bisector. The angle is labeled *A, B,*and C, and the bisector is labeled *A* and *D.*

*In the observational experiment,* the subject at first says there is a bisector. “If we measure it with a protractor... No... we have to measure it first with a protractor.” The subject measures it, sees that the angles are not equal, decides that it is not a bisector. She reads the problem conditions again, which say that the straight line divides the angle into two equal parts, and says that it only divides the angle *DAC* into equal parts.

Experimenter: Where are the equal parts?

Subject: Side *AB* is equal to side *AD,* but side *AC* is not equal to side *AD,* so the straight line is not a bisector.

We see that the subject cannot escape the confusion created by the discrepancy between the problem conditions and the illustration. The subject readily concludes that there is no bisector, easily changes its attributes.

*In the training experiment,* the subject approaches the solution to this problem like this: after reading the problem to herself, she quickly begins to recite the problem conditions aloud. The experimenter interrupts: “Have you solved the problem?”

Subject: Yes, the illustration is not for this problem: the problem is about a bisector, but that’s not in the illustration.

Experimenter: Why did you think it was a bisector?

The subject correctly identifies the attributes of a bisector, shows that they are present in the problem conditions, and adds: “In the illustration, the line has in common with the bisector that the line comes from the vertex, but it does not bisect the angle.” She quickly measures it with a protractor: “One is 40^°^, the other is 10°.”

The same thing occurred when this subject was solving other problems: helplessness in the observational experiment, but confident, quick, and correct solution of the same problems in the training experiment.

Subject K. solved the problems in the training experiment just as easily. As an example, we give her solution to *Problem No. 6.*

The problem conditions describe a straight line that divides a given angle in a ratio of 3:8. The subject has to establish whether this line is the bisector of the given angle. An illustration was provided, in which a straight line is placed in the position of the bisector.

The subject reads the problem and quickly says: “This won’t be a bisector.”

Experimenter: Why?

Subject: Because the ratio is 3:8. It would have to be 3:3 or 8:8.

Experimenter: Are there any attributes of a bisector?

Subject: Yes. An *angle* is given, with a *straight line* drawn inside it.

Experimenter: What attribute is missing?

Subject: That it divides this angle into two equal parts.

Note that the discrepancy between the illustration and the problem conditions does not bother this subject either.

Thus we see that the necessary and sufficient attributes are clearly identified by both subjects; they use them correctly even when the illustration contradicts the problem conditions, which had previously led subject N. to complete confusion. Since we did not do any additional work with the concept of “angle bisector” with subject N., and subject K. was basically dealing with this concept for the first time, this can only be explained as follows: when forming the concepts of “straight line” and “angle,” the subjects learned *a certain method of acting with these concepts, which they transferred to the concept of “angle bisector.”* In other words: *there was a generalization of the method of action with the attributes of concepts of this type.*

In subject N. (sixth grader), such a transfer was also found for the concepts of “adjacent angles,” “supplementary angles,” and “perpendicular.” When establishing the concepts of adjacent and supplementary angles, as well as when establishing the bisector and the perpendicular, the subject immediately took the right path, skillfully using the attributes of the concept, but she only solved the easiest problems in her head, whereas for more difficult problems, she pronounced the attributes aloud, then searched for them aloud in the problem conditions.

So, when solving *Problem No. 8,* in which two angles were presented that had a common vertex and a common side, and it was required to determine whether the angles would be adjacent, the subject reasons aloud as follows: “Given two angles, they have a common vertex, a common side ... We know that they are adjacent when there are two angles, a common vertex, and a common side. That’s three.”

Experimenter: Have you made up your mind?

Subject: No.

Experimenter: Well, please continue.

Subject: But the problem conditions doesn’t say that the side is between the others. According to the statement they are not adjacent, but according to the illustration they are. But we have to rely on the problem conditions (the problem is solved correctly).

We see that the subject approaches the task confidently, immediately takes the right path, but solves it only when she works through it aloud. Obviously, the transfer at a lower level in this case is explained by the fact that the concepts of “adjacent angles” and “supplementary angles” include many attributes compared to the bisector and the perpendicular, and these attributes are more difficult to pick out in the problem conditions.

With subject K., we did not check the transfer to other concepts, except for the bisector.

The phenomenon of transfer during the formation of geometric concepts was also described in a University student’s work performed under our guidance.

These results provide a basis to think that with careful stage-by-stage development of the elementary concepts, during which the method of action with the attributes of these concepts is also mastered, formation of the basic system of geometric concepts will not be difficult and will proceed faster, without careful development in stages.

In the formation of concepts through action with objectified attributes, another series of facts was discovered, also related to *generalization of concepts,* but of a different kind. If the facts we are looking at suggest generalization occurring within *a system of concepts,* then these facts also suggest generalization occurring within *one concept.*

The first type of generalization is generalization of *the method of action* with concepts; the second, which we now discuss, is *generalization of the concept according to the material.*

This type of generalization was shown in the fact that, with our method of training, the subjects were not bound by particular features of the illustration, as is usually the case in school assignments. Specifically, this was expressed as follows:

First, when solving problems, the subjects were not only not bothered, but were not even surprised by the unusual position of the illustration.

Second, the subjects, at the experimenter’s request, readily presented different versions of the illustrations. So, for example, after introducing the definitions of right, obtuse, and acute angles, and of the perpendicular, the experimenter asked subject N. (sixth grader) to present different acute, right, and obtuse angles, and different perpendiculars. The subject presented them in quite varied spatial configurations.

Third, even after the same spatial position was deliberately presented many times, a quite different position of the figure did not cause difficulties. So, when forming the concept of “straight line” in subject K. (fifth grader), we deliberately presented only the horizontal position of the straight line. A preliminary interchange showed that the subject only calls horizontal and vertical lines “straight lines,” and calls oblique lines “slanted.” “It’s not straight,” says the subject. We deliberately did not correct her. During the training, a horizontal line was also drawn on a card along with the attributes. The first six problems were presented with horizontal lines. The subject tested all of them with a thread. In the seventh problem, a vertical line was shown, and in the eighth problem, an oblique line. The experimenter asked the subject to say what type of line it was, without reading the problem and without using a thread. The subject quickly and confidently answered that it was a straight line. When the experimenter asked why she thought that, the subject replied, “It will align with a taut string.” Thus, in the subject’s definition of a straight line, only alignment with the thread matters, i.e., the attribute that had been presented to her, which she had worked with. After that, she was given an array of straight lines, in the most diverse spatial positions. The subject just as confidently answered that they were all straight lines.

It should be noted that even after a long break, freedom from the particulars of the illustration is preserved. Thus, five months after subject N. (sixth grader) had solved problems that required the use of the concept of “perpendicular,” we gave her a problem that asked how to find out which lines in the illustration are perpendicular. The illustration shows four perpendiculars in unusual positions and one oblique line in the normal position, the oblique line having a very small angle of inclination. The subject gave the following answer: “You need to measure it; where there is a right angle, there is a perpendicular.” When the experimenter asked which lines seemed to her to be perpendicular, the subject identified all four correctly; as for the oblique line, she said that it would not be perpendicular.

These results give us reason to think that, under certain conditions, a concept can be generalized even without variations in the illustration.

Apparently, students’ well-known difficulties when encountering an illustration in an unusual position, which suggest insufficient generalization of concepts, are explained by the particular ways in which they have learned. In school assignments, students are not, as a rule, given the attributes of a concept in an objectified form; they are not taught to use these attributes for specific tasks. By no means everyone can do it on their own. For that reason, in a significant proportion of students, the definition of a concept that can be reproduced by them without error appears “not to be working,” and the illustration that was given to them when the concept was defined spontaneously becomes the actual reference point. When trained in this way, a large number of variations of the illustration becomes necessary for the gradual identification and generalization of the attributes of the concept.

That is a very good confirmation of the fact that when operating with geometric concepts, a visual image often serves as a reference point. In geometry, adjacent angles are defined as angles that have a common vertex and a common side. We also introduced this definition in the training experiment. But since we asked our subjects to use the attributes of the concept, here is what happened: subject N. (sixth grader) told us that the angle and the part of this angle adjacent to one of its sides were adjacent. When we tried to object, she replied that they fit with the attributes presented, and we had to agree, and then introduce an additional attribute: the common side has to be located between the other sides of the angles.

This suggests that not only students, but also those who teach them and write textbooks for them, sometimes think visually in practice, and not applying the attributes of a concept.

Since from the very beginning we presented the attributes in a single, generalized, and objectified form and taught the subjects to use them as criteria for what to look for in the problem conditions, rather than in an illustration of specific problems, the subjects were freed from the constraint of particular features of the illustration. It should be noted that it also may have mattered that the attribute itself was not established when the concept of “straight line” was formed: the subject was free to pull the thread into different spatial configurations. This could be decisive for generalization of the concept of “straight line.” Independence from the specifics of the illustration in the application of other concepts might already have been the result of a transfer. So far, this is only one possible conjecture, needing factual verification.

So, the essence of the results we have presented is as follows:

1. With the stage-by-stage working out of several elementary concepts, the method of action is mastered along with the concepts themselves, and is transferred to subsequent concepts, which therefore can be formed immediately at the level of already achieved skills.
2. Provided that the attributes of a concept are not stated in only a generalized form, but are also given objectively and applied as criteria for the presence or absence of the relevant phenomenon, they are mastered from the very beginning in a generalized way, and their generalization by varying the material becomes superfluous.

